# *In Situ* Luminescence of Self-Assembled Eu(III)-Naphthoic Acid Complex in Langmuir and LB Films

**DOI:** 10.1021/acs.langmuir.5c03763

**Published:** 2025-10-09

**Authors:** Sofia Sestito Dias, Maria Izabel Xavier Scapolan, Wilson Aparecido de Oliveira, Rhayane Margutti Rocha, Higor Henrique de Souza Oliveira, Marian Rosaly Davolos, Eduard Westphal, Renata Danielle Adati

**Affiliations:** † Academic Department of Chemistry and Biology, 74354 Universidade Tecnológica Federal Do Paraná (UTFPR), Curitiba, PR 80230-901, Brazil; ‡ Department of Analytical, Physical-Chemistry and Inorganic Chemistry, 153997 São Paulo State University (Unesp), Institute of Chemistry, Araraquara, SP 14800-060, Brazil; § Department of Chemistry, Universidade Federal de Santa Catarina, Florianópolis 88040-900, Brazil; ∥ IFSP - Instituto Federal de Educação, Ciência E Tecnologia de São Paulo, IFSP - Campus, Matão, SP 15991-502, Brazil

## Abstract

Highly luminescent nanostructured films with controlled molecular organization may find applications in various areas, ranging from new drug delivery systems to solid-state lighting. In this work, we synthesized and characterized amphiphilic complexes [Ln­(dion)_3_(H_2_O)­(DMSO)] where Ln­(III) = Eu or Gd and dion is 6-dodecyloxy-2-naphthoic acid. The ligand dion was prepared in high yield through a sequence of esterification, alkylation, and hydrolysis reactions, and its structure was confirmed by ^1^H NMR analysis. The synthesis of the complexes was confirmed by CHN elemental analysis and FT-IR spectral data. The triplet level (*T* = 26,332 cm^–1^) obtained from an isostructural Gd­(III) complex confirms that the dion ligand acts as an antenna in the energy absorption and transfer process. The photoemission spectra exhibit intraconfigurational transitions from the Eu­(III) ion, with the hypersensitive transition ^5^D_0_ → ^7^F_2_ at 623 nm being the most intense line. An intrinsic quantum yield $\left(Q_{Eu}^{Eu}\right)$ of 40.45% indicates that the long chain of dion (C_12_) causes losses by nonradiative processes, in addition to the low value of the Ω_2_ parameter, characterizing a lower covalence of the chemical coordination environment. Luminescent monolayer films were obtained through the complexation that occurred at the interface, and the photoemission was monitored *in situ* using a Langmuir trough. The results indicate that the dion carboxylic acid-Eu­(III) interfacial film exhibits f–f intraconfigurational transitions. Therefore, the behavior of the molecularly organized films demonstrates that dion plays the roles of both surfactant and photoantenna, enabling the deposition of stable and luminescent Langmuir–Blodgett (LB) films.

## Graphical abstract

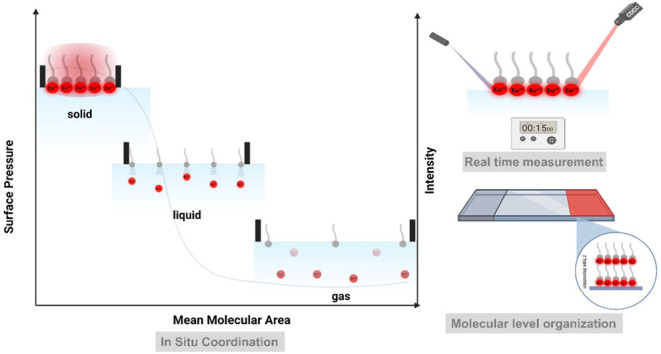

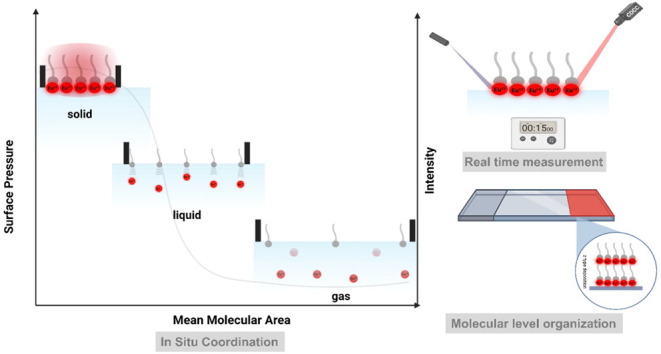

## 1 Introduction

Lanthanide, Ln (III) ions are attractive owing to their distinctive photophysical characteristics, stemming from the shielded 4f-electron configuration. Such properties include sharp emission bands, long excited-state lifetimes, large Stokes shifts, and high color purity,^1,2^ which make lanthanide-based complexes promising for devices, sensors, and optoelectronic applications. Despite these advantages, Ln­(III) ions have a low absorption coefficient for the 4f–4f transitions, classified as prohibited transitions by Laporte’s rule.^1,3^ To overcome this condition, organic ligands are often employed to increase the absorption coefficients of Ln­(III) complexes through the π–π* transition of organic moieties, thereby enhancing the lanthanide luminescence.^3,4^ Therefore, through this mechanism, the 4f–4f emission is induced by the transfer of excitation energy via intersystem crossing, resulting in highly luminescent lanthanide complexes that can be tailored according to the energy of the donor state ligand.^3^

Coordinating Ln­(III) ions with carboxylate units allows the formation of varied complexes with tunable structures and properties.^5–9^ Strategies to enhance the luminescent and structural characteristics of europium complex films with amphiphilic ligands have been explored by studying amphiphilic naphthalene-based derivatives, known for their high absorption coefficients, which show promise as efficient antenna ligands to enhance Eu­(III) emission for applications in sensing and probing.^10,11^ The molecular structure of amphiphilic ligands supports the formation of two-dimensional coordination systems, with the hydrophobic region ensuring water insolubility and the hydrophilic part anchoring the molecules at the interface. Research in this area has provided valuable insights into the design required for ligands used in thin-film applications, including long-chain carboxylic acids, fatty acid phosphate esters, and β-diketonates.^10–16^

In most cases, emissive materials are not applied in solution but rather in the solid state, typically as thin films. Among the various techniques for fabricating molecular thin films, wet-processing methods based on bottom-up strategies are particularly valuable. In particular, the Langmuir–Blodgett (LB) technique enables the sequential combination of different materials to form well-organized and structurally defined architectures.^17–19^ Furthermore, from a fundamental perspective, Langmuir monolayers offer an ideal platform to investigate molecular interactions under controlled interfacial organization, leading to applications such as molecular electronics, sensors, biosensors, and drug delivery.^14,15,20–22^

LB films of Eu­(III) complexes allow the transfer of organized monolayers onto solid substrates, preserving or even enhancing their luminescent properties. Compared to their solution or powder counterparts, these films often exhibit enhanced emissions from the ^5^D_0_ and ^5^D_1_ excited states, particularly when nonsymmetrical β-diketonate or long-chain amphiphilic ligands are employed.^4,12,17^ Such organized systems can also exhibit linearly polarized luminescence (LPL), which is advantageous for optical devices that require directional light emission.^4,10^ Importantly, *in situ* complexation at the air–water interface enables the direct formation of Eu­(III)-ligand assemblies during film fabrication, as evidenced by surface pressure–area isotherms and real-time photoluminescence spectroscopy.^4,10,18^ The molecular ordering induced by the amphiphilic nature of the ligands and hydrophobic chain interactions contributes to the reduction of nonradiative deactivation, maintaining the electronic integrity of the lanthanide center.^3,4,10^

In this context, this study aims to design a ligand that functions as an effective antenna for complexation with Eu­(III), while also enabling the formation of well-organized Langmuir and Langmuir–Blodgett (LB) films for the investigation of optical properties. Real-time photoluminescence measurements were performed during the interfacial complexation process, in which a long-chain amphiphilic ligand derived from naphthoic acid (dion, C_12_) was spread at the surface of an aqueous subphase containing europium nitrate in a Langmuir trough.

## 2 Experimental Section

### 2.1 Materials and Methods

All reagents and solvents employed in the synthesis were of analytical grade and used as received. Solutions of Eu­(III) and Gd­(III) nitrates were prepared by dissolving their respective oxides (Sigma-Aldrich 99.9%) in concentrated nitric acid and diluting them with distilled water. Triethylamine (99.8%) was purchased from Reagen, nitric acid (65%) was from Alphatec, and ethanol (95%) was from Neon. All solvents were of analytical grade and used without prior purification.

The chemical stoichiometries of the complexes were suggested by Ln­(III) titration using a 0.01 mol L^–1^ ethylenediaminetetraacetic acid (EDTA) solution and elemental analysis (PerkinElmer CHN 2400). IR spectra in the 4000–400 cm^–1^ region at a resolution of 4 cm^–1^ were recorded using a Varian 640-IR infrared spectrometer by the conventional KBr method.

### 2.2 Experimental Procedures and Characterization Techniques

#### 2.2.1 Synthesis of the 6-Dodecyloxy-2-naphthoic Acid (Dion) Ligand

The synthesis of dion was carried out following a previously adapted procedure.^19^ The stages of the synthetic route are represented in Figure 1, beginning with the Fischer esterification of the carboxylic acid. In this step, 1.50 g (8 mmol) of 6-hydroxy-2-naphthoic acid (**1**), 30 mL of ethyl alcohol, and 0.2 mL of concentrated sulfuric acid were mixed. The reaction mixture was refluxed under stirring for 18 h. After completion, the solvent was removed under reduced pressure using rotary evaporation. The resulting solid was dissolved in 30 mL of ethyl acetate, washed with distilled water, and dried over anhydrous sodium sulfate. The solvent was then evaporated to yield a solid (**2**), which was used in the next step without further purification.

**Figure 1:**
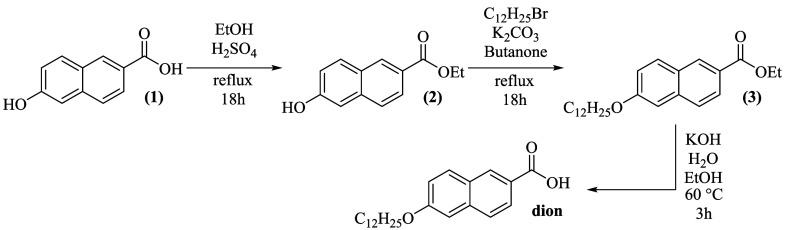
Route to the synthesis of 6-dodecyloxy-2-naphthoic acid (dion).

In the alkylation step, ethyl 6-hydroxy-2-naphthoate (**2**) (1.44 g, 6.66 mmol) was placed in a round-bottomed flask and mixed with K_2_CO_3_ (1.49 g, 10.8 mmol), C_12_H_25_Br (2.02 g, 7.98 mmol), and 50 mL of methyl ethyl ketone. The mixture was refluxed under stirring for 18 h. Subsequently, the reaction mixture was filtered to remove insoluble solids, and the solvent was evaporated, resulting in the alkylated product **3**, which was used directly in the next step without further purification.

In the final step, the ester group of compound 3 was hydrolyzed by treatment with KOH (1.34 g, 23.9 mmol) in a mixture of ethanol (40 mL) and water (20 mL) at 60 °C for 3 h. After that, the solvent was partially removed, the residue was diluted with water, and the pH of the solution was adjusted to 1–3 to precipitate the product. The solid was then filtered, washed, and recrystallized from ethanol/water to yield the purified ligand (dion).

The pure white crystalline solid of dion was obtained in a 620 mg yield (29% overall). Its structure and purity were confirmed by ^1^H NMR spectroscopy, recorded on a Bruker AVANCE DRX 400 spectrometer operating at 400 MHz, and it is consistent with the literature data.^23^

Melting point (liquid crystal) Cr 118 °C SmC 150 °C N 168 °C Iso. ^1^H NMR (400 MHz, CDCl_3_) δ ppm = 8.56 (d, *J*_4_ = 1.6 Hz, 1H, Ar–H), 8.05 (dd, *J*_3_ = 8.7 Hz, *J*_4_ = 1.6 Hz, 1H, Ar–H), 7.84 (d, *J*_3_ = 8.9 Hz, 1H, Ar–H), 7.74 (d, *J*_3_ = 8.7 Hz, 1H, Ar–H), 7.18 (dd, *J*_3_ = 8.9 Hz, *J*_4_ = 2.4 Hz, 1H, Ar–H), 7.15 (d, *J*_4_ = 2.4 Hz, 1H, Ar–H), 4.09 (t, *J*_3_ = 6.5 Hz, 2H, OCH_2_^–^), 1.86 (m, 2H, OCH_2_CH_2_^–^), 1.51 (m, 2H, OCH_2_CH_2_CH_2_^–^), 1.43–1.21 (broad signal, 16H, −CH_2_^–^), 0.88 (t, *J*_3_ = 6.3 Hz, 3H, −CH_3_) (Figure S1).

#### 2.2.2 Synthesis of [Ln­(dion)_3_(H_2_O)­DMSO] Complexes

The [Ln­(dion)_3_(H_2_O)­DMSO] complexes were prepared following a literature-adapted method.^4^ Europium oxide (5.1 × 10^–5^ mol) was dissolved in nitric acid (40 μL) to form Eu­(NO_3_)_3_·6H_2_O, which was then dissolved in 14 mL of ethanol. This solution was slowly added to a mixture of dion (5.0 × 10^–4^ mol) and triethylamine (350 μL). After approximately 23 h under stirring, the formation of a precipitate was identified. The resulting fine white solid was filtered and washed with cold water and DMSO/THF to remove spurious material, and then dried to obtain a final product with a yield of 97%. The solid displayed red emission under a UV lamp, 365 nm. The same procedure was carried out to obtain the gadolinium analog complex from the precursor Gd_2_O_3_·6H_2_O (1.02 × 10^–4^ mol) (93%) to estimate the excited triplet energy level (*T*_1_). Microanalysis measurements were made, and the results suggest the presence of H_2_O and DMSO, resulting in theoretical values that match the experimental ones (experimental/calculated, %): (%C 64.87/64.46; %H 7.74/7.60; and %Eu­(III) 11.55/11.13, %C 64.86/64.78; %H 7.74/7.57; and %Gd­(III) 11.96/10.61). The recorded elemental data closely matched the calculated values, suggesting the formation of a complex with the stoichiometric formula [Eu­(dion)_3_(H_2_O)­DMSO] (see Figure 2) corresponding to the molecular formula C_71_H_101_O_11_SEu.

**Figure 2:**
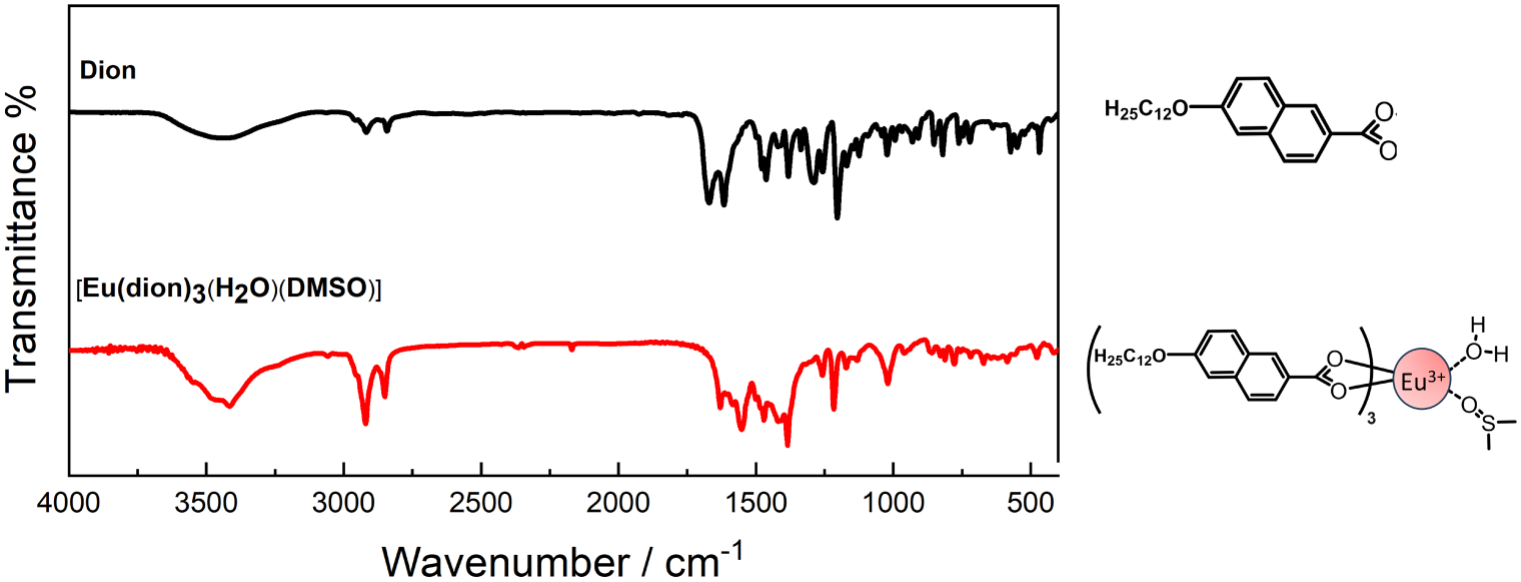
Infrared spectra using KBr pellets and chemical structures of the dion and [Eu­(dion)_3_(H_2_O)­(DMSO)] complex.

### 2.3 Photophysical Characterization

The emission and excitation spectra of the bulk complexes were recorded in a Horiba-Jobin Yvon Fluorog-3 FL3–222, spectrofluorometer equipped with a Hamamatsu R928P photomultiplier. A 450 W continuous Xenon Short Arc Lamp (UXL-450S-O, USHIO INC.) was employed for excitation and emission spectra, while luminescence decay curves utilized a 0.15 J per flash High-stability Short Arc Xenon Flashlamp (FX-1102, Excelitas Technologies), with an initial delay of 0.05 ms. To determine the emission lifetime, emission decay curves were monitored at 273 nm, considering the excitation on the ligand band, and the emission at the ^5^D_0_ → ^7^F_2_ transition of the Eu­(III) ion, using a pulsed xenon 450 W lamp. The phosphorescence spectrum of the isostructural gadolinium complex was employed to determine the *T*_1_ energy level of the dion, utilizing the tangent line drawn on the first Gaussian curve obtained by deconvoluting the spectrum (zero-phonon). This spectrum was acquired in powder form at 77 K.

### 2.4 Preparation and Characterization of Langmuir Monolayers

Ultrapure water obtained in a Merck Millipore Direct-Q 3 water purification system composed the subphase. Owing to the luminescent monolayer, the subphase was saturated with Eu­(NO_3_)·6H_2_O (2 × 10^–5^ mol L^–1^). The Langmuir–Blodgett KSV Minitrough was used to investigate all films at 298 K. The surface pressure as a function of the available area per molecule (isotherms: π × *A*), surface pressure stability tests, and the reversibility of the self-organization process during compression and expansion of barriers (hysteresis test) were studied.

To ensure a proper dispersion, monolayer formation, and reproducibility of experiments, after the dispersion of the dion solution, 20 min were waited to ensure the complete evaporation of the dichloromethane and to evaluate the formation of the complex at the interface (film spread in a saturated subphase). Then, compression of the monolayer began by simultaneous and symmetrical closure of the barriers at a constant rate of 10 mm min^–1^.

*In situ* excitation was performed using an optical fiber (200 μm core, 240–1200 nm, 1.8 m) coupled to an ISS illuminator, model P110, equipped with a monochromator (focal length of 100 mm, resolution of 1.0 nm, F/3.5 aperture, 32 × 32 mm diffraction grating) and a 300 W xenon continuous arc lamp (230–850 nm).

The *in situ* detection of the photoluminescence of the Langmuir films was performed using an optical fiber (SR-OPT-8024, one-way fiber bundle, 200 μm core, HOH-UV/vis, 2.0 m) in a front-to-back configuration. A face coupled spectrophotometer SHAMROCK 303i, Andor Tech., with a diffraction grating of 600 lines mm^–1^ and a CCD camera detector NEWTON DU940P-BV, Andor Tech., with 2048 × 512 pixels) was used. The detection fiber is kept at an angle of 22.5° from the excitation fiber.

### 2.5 Deposition of Langmuir–Blodgett (LB) Films

The glass substrates used for LB film deposition (LB4) were pre-cleaned through a multistep procedure involving immersion in a 5% (v/v) aqueous solution of Extran in an ultrasonic bath at 80 °C for 10 min, followed by thorough rinsing with distilled water. Next, the substrates were immersed in isopropyl alcohol at 80 °C for 10 min and then dried in an oven at 100 °C for 30 min. The LB films were formed by Z-type deposition after allowing 20 min for the ligand solution to disperse on the water surface, with a fixed surface pressure of 15 mN m^–1^, a barrier closing speed of 30 mm min^–1^, and dipper displacement controlled by descent and ascent speeds of 70 and 10 mm min^–1^, respectively. This Z-type deposition resulted from the transfer of the dion film occurring exclusively during substrate immersion, which was influenced by the ligand’s molecular orientation and favored Z-type over Y-type film formation.

The LB films were characterized by photoluminescence with ultraviolet excitation (UV-PLS) in a Fluorolog Horiba Jobin Yvon spectrofluorimeter, model FL3-222, in a front-face configuration (detection angle concerning the excitation equal to 22.5°) and using a 450 W continuous xenon lamp as an excitation source.

## 3 Results and Discussion

### 3.1 Lanthanide Complexes

#### 3.1.1 Infrared Spectral Analysis

Figure 2 presents the comparative IR spectra of the dion and its corresponding europium complex [Eu­(dion)_3_(H_2_O)­(DMSO)]. The intense band in the region of 1681 cm^–1^ in the ligand spectrum refers to the symmetrical stretch ν_s_(C=O), while for the complex, this band is found around 1635 cm^–1^. These data confirm the weakening of the C=O bond, as there is a partial shift in the carbonyl π electron density to form the bond between the metal and oxygen, as already observed by Yoshihara and collaborators.^4^ Therefore, the lower stretching frequency attributed to the complex confirms the coordination of the ligand to the metal ion.^24^

The O–H stretching of the carboxylic acid is observed at 3448 cm^–1^ and overlaps with the C–H stretching of the aromatic ring of the naphthyl group. In the Eu-complex spectrum, the broad band in the same region of 3448 cm^–1^ also refers to O–H stretching of water molecules.^24–26^ The band around 1018 cm^–1^ is attributed to the S=O stretching of DMSO coordinated with Eu­(III) ions, which normally appears at ∼1024 cm^–1^ for free DMSO molecule vibration. The shift to a lower wavenumber indicates a metal ion coordination through the oxygen atom.^22,27^ The main attributions of the vibrational modes of [Gd­(dion)_3_(H_2_O)­(DMSO)] are similar to those of the Eu­(III) complex, both are shown in Table S1.

#### 3.1.2 Photoluminescence Spectroscopy

Owing to the close similarity in the ionic radii of Gd­(III) (1.107 Å) and Eu­(III) (1.120 Å) ions,^28^ the gadolinium complex is commonly employed as a chemical analog to simulate the coordination environment of Europium­(III). This is useful for elucidating the energy level structure of coordinated ligands, with an emphasis on determining the triplet (*T*_1_) energy levels. The emission spectrum of the [Gd­(dion)_3_(H_2_O)­(DMSO)] complex was used to determine the triplet level of the dion. The emission state energy value was defined as *T* = 26,332 cm^–1^ as shown in Figure S2. The higher triplet relative to the emitting level of Eu­(III) (17,250 cm^–1^) provides evidence that dion acts as an antenna in the ion sensitization mechanism.

The excitation spectrum of the [Eu­(dion)_3_(H_2_O)­(DMSO)] complex was registered at room temperature and monitored at λ_em_ = 614 nm, the hypersensitive transition of Eu­(III). Narrow lines are assigned to the intraconfigurational transitions of Eu­(III) ^5^D_4_ (360 nm), ^5^L_6_ (393 nm), ^5^L_7_ (375 nm), and ^5^D_2_ (464 nm) in addition to absorptions at ∼273 and 330 nm attributed to the dion ligand, as shown in Figure 3a. The broad and intense absorption bands at ∼280 nm are associated with the intraligand transition states S_0_ → S_1_ and the transfer of energy from the ligand to the metal; as observed, the spectral profile indicates that the excitation of the ligand via LMET (ligand-to-metal energy transfer) results in the emission of Eu­(III) ions. Therefore, there is a transfer of energy from the dion to the excited states of the europium ion.^28–30^

**Figure 3:**
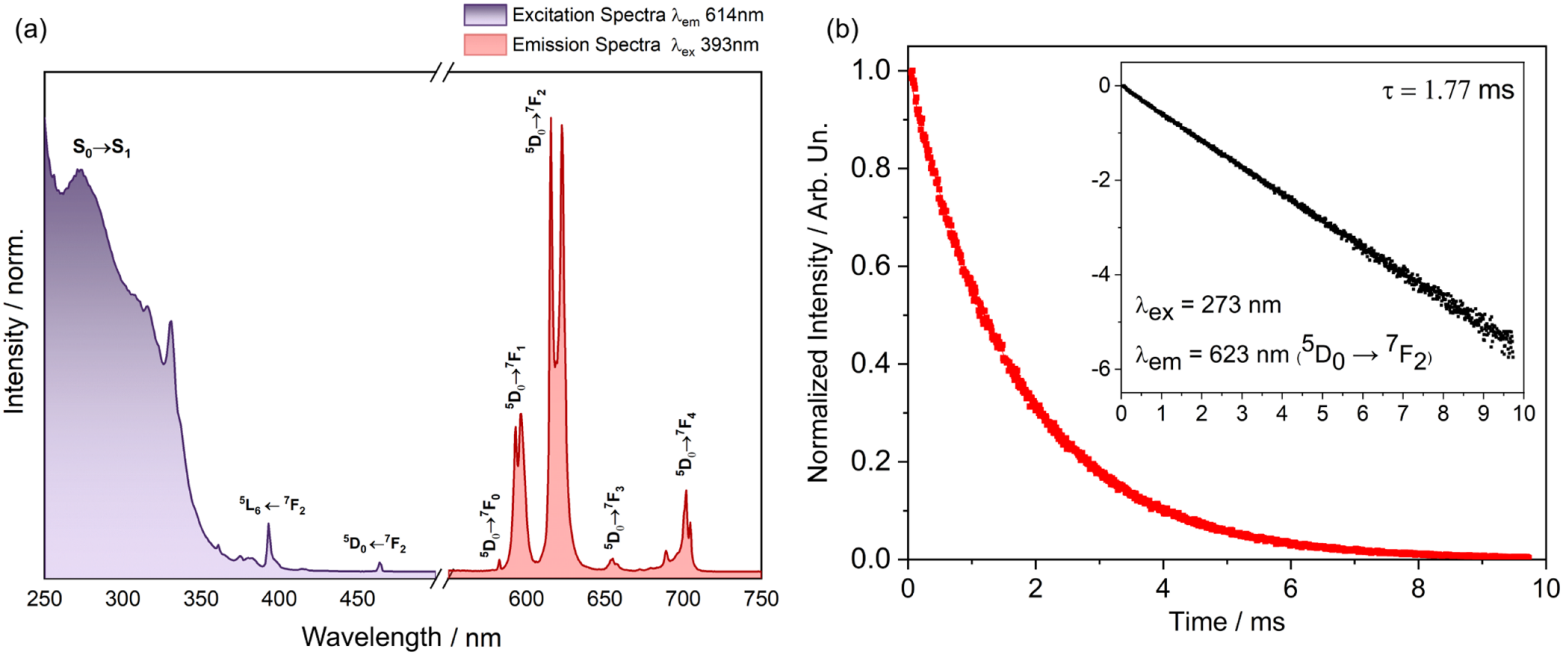
(a) Excitation and emission spectra of the [Eu­(dion)_3_(H_2_O)­(DMSO)] complex at 298 K at λ_em_ = 614 nm and λ_exc_ = 393 nm. (b) Emission decay curve at room temperature monitoring the ^5^D_0_ → ^7^F_2_ transition at λ_exc_ = 273 nm.

Figure 3a shows the emission spectra of the [Eu­(dion)_3_(H_2_O)­(DMSO)] complex under excitation at λ_exc_ = 393 nm. The spectrum reveals characteristic intraconfigurational transitions of the europium­(III) ion ^5^D_0_ → ^7^F_J_ (*J* = 0, 1, 2, 3, 4), with the most intense emission being that of the hypersensitive transition ^5^D_0_ → ^7^F_2_ (hypersensitive transition) in 614 nm, which compared to the ^5^D_0_ → ^7^F_1_ transition (MD allowed, not sensitive), this suggests that the Eu­(III) ion is situated in an environment without an inversion center, indicating that the compound has low symmetry.^29^ The same emission spectra profile was observed when monitored when excited with different wavelengths (λ_exc_ = 273, 330, and 393 nm). The maximum number of lines of the forbidden transition ^5^D_0_ → ^7^F_0_ = 1 is identified, proportional to the number of sites (without a center of inversion) occupied by the europium ion.

The lifetime of the emitter level (τ) of approximately 1.77 ms was determined when excitation occurred at 273 nm (LMCT), considering the emission at 623 nm. The decay mode from lifetime measurements indicates the presence of a single emissive site (Figure 3b), as the data fit a first-order exponential function. Consistent with the photoemission results, this single site is assigned to the ^5^D_0_ → ^7^F_0_ transition, characteristic of only one determined lifetime value. Comparatively, the lifetime values and quantum efficiency are justified by nonradiative processes, which depend on the number of carbon atoms in the counterions and β-diketones, due to the multiphonon relaxation associated with the CH vibrational groups.^29–31^

##### 3.1.2.1 Luminescence Efficiency and Judd–Ofelt Analysis

The emission spectrum and experimental lifetime were used to obtain the Judd–Ofelt intensity parameters (Ω_λ_).^31–33^ The *A*_rad_ and *A*_nrad_ values refer, respectively, to radiative decay rates and nonradiative decay rates, τ is designed to the experimental emission lifetime of the ^5^D_0_ level and $Q_{Eu}^{Eu}$ the intrinsic quantum yield. The methodology used to calculate the intensity parameters Ω_2_ and Ω_4_ and *R*_02_ is detailed in the Supporting Information.

The intensity parameters indicate structural changes in the coordination environment and the character of the covalent bond between the ligand and the metal ion. The Judd–Ofelt intensity parameter Ω_2_ is highly sensitive to the asymmetry of the ligand field surrounding the Eu­(III) ion, providing insight into the local coordination environment. In contrast, the Ω_4_ parameter is associated with the rigidity of the system and is also influenced by the point symmetry around the Eu­(III) ion.^29–32^ As reported in the literature, similar to the dion ligand, the long alkyl chain (C_12_) is the main factor influencing the covalence of the Eu­(III) complex since it induces a steric effect that may promote molecular folding and alter the local symmetry, as suggested by the low value of Ω_2_.^31^

Moreover, the presence of extended hydrophobic chains can promote a more rigid and ordered supramolecular arrangement, which may indirectly influence the vibrational coupling and crystal field effects experienced by the Eu­(III) ion. These long-range interactions can modify the vibrational modes or reduce the dynamic flexibility of the system, leading to a decrease in the Ω_4_ parameter.^31^ The results are aligned with previous studies on DMSO-based systems, where lower vibrational frequencies reduce nonradiative deactivation and extend excited-state lifetimes. Sulfoxide-based ligands in Eu­(III) complexes enhance quantum yields by shielding the coordination environment, while improving volatility and charge transport, making them promising candidates for Light Conversion Molecular Devices (LCMDs) and electroluminescent applications^31–33^ (Table 1).

**Table 1:** Experimental Photophysical Parameters (Judd–Ofelt Parameters (Ω_2_ and Ω_4_), Radiative (*A*_rad_) and Nonradiative Decay Rates (*A*_nrad_), Luminescence Lifetime (τ), Intrinsic and Quantum Yield $\left(Q_{Eu}^{Eu}\right)$ ), Calculated from the Experimental Emission Spectrum of [Eu­(dion)_3_(H_2_O)­(DMSO)] Complex

Structure	Ω_2_ (10^–20^ cm^2^)	Ω _4_ (10^–20^ cm^2^)	*A*_rad_ (s^–1^)	*A*_nrad_ (s^–1^)	τ (ms)	$Q_{Eu}^{Eu}$ (%)
[Eu(dion)_3_(H_2_O)(DMSO)]	5.40	1.21	336.46	564.97	1.77	40.45

### 3.2 Langmuir Monolayers and LB Films Studies

Figure 4a,b shows π × *A* isotherms of the dion ligand at the interface of water (a) and an aqueous solution of europium nitrate at 2.0 × 10^–5^ mol L^–1^. The structuring of the film is a result of the greater interaction of the alkyl portion of the dion ligand, and consequent formation of aggregates. For dion ligand isotherms at water or an aqueous solution of europium nitrate, the collapse region is near 33 mN m^–1^, confirming that the approximation between the alkyl chains (C_12_) is determinant in the condensed phase (C) for the monolayer organization, revealing greater interaction and packing of the hydrophobic portion of the chains, until the effective collapse of the films. Notable isotherm changes are observed to the liquid-expanded (LE) to liquid-condensed (LC) phase transition region, particularly in the molecular areas that vary depending on the subphase composition. Figure 4b also shows that the transition from the gaseous-liquid phase occurs at a higher molecular area for the europium nitrate solution (84.6 Å^2^ molecule^–1^) than for water (14.7 Å^2^ molecule^–1^) in the subphase, confirming the coordination of the carboxylate groups and Eu­(III) ions from the europium nitrate–water interface. The higher Cs^–1^ values obtained for the saturated subphase further corroborate this behavior, evidencing stronger subphase–molecule interactions and resulting in a more rigid and less compressible monolayer. In contrast, the lower Cs^–1^ values in pure water confirm the formation of a more compressible and flexible film, consistent with weaker interactions in the absence of europium ions. The compressibility modulus Cs^–1^ of the dion ligand at the water subphase and at an aqueous solution of europium nitrate at 2.0 × 10^–5^ mol L^–1^ is presented in Figure S3.

**Figure 4:**
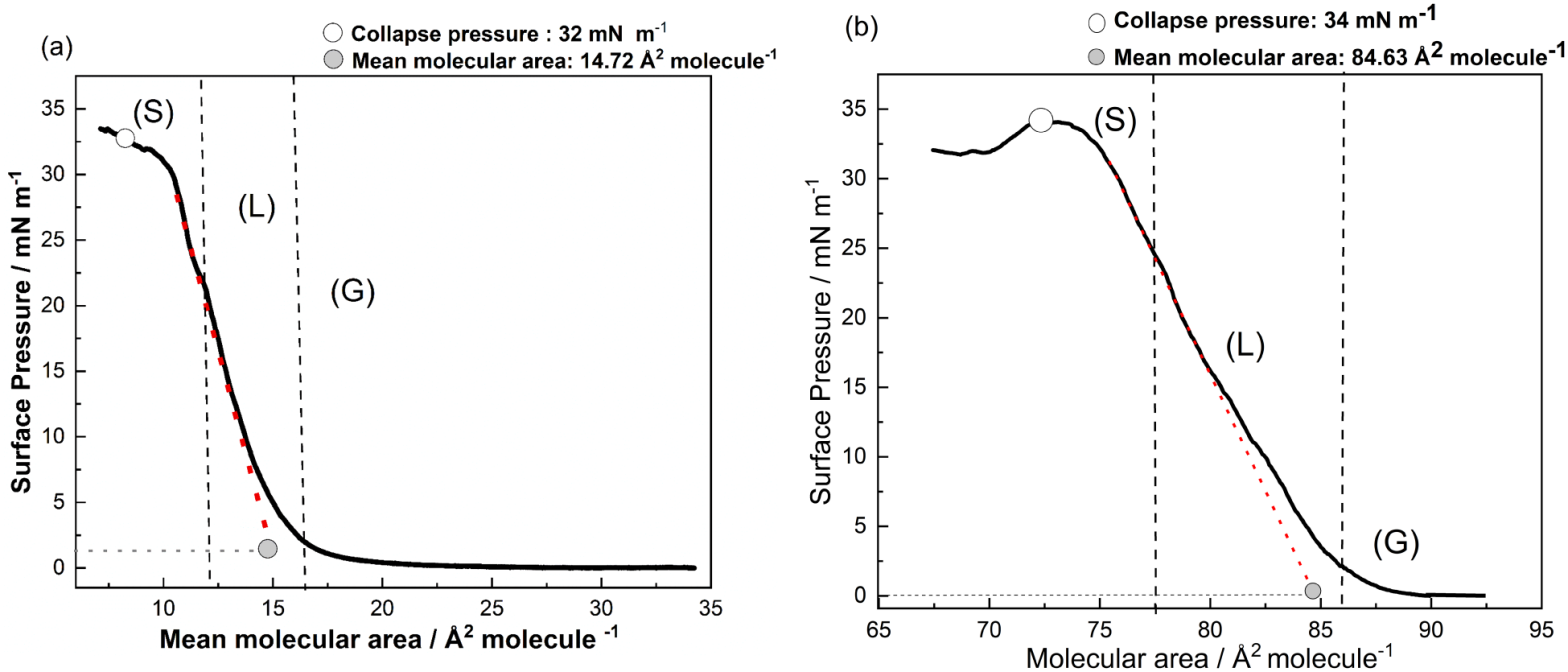
π × *A* isotherms of surface pressure versus molecular area of the dion ligand at (a) water and (b) an aqueous solution of europium nitrate at 2.0 × 10^–5^ mol L^–1^.

The stability tests of the dion monolayer at the water surface (Figure 5a) and at an aqueous solution of europium nitrate at 2.0 × 10^–5^ mol L^–1^ (Figure 5b) were monitored over 4000 s, respectively under the initial surface pressure of 17 or 15 mN m^–1^. Accordingly, on water, 1300 s is necessary for the barriers to reach the desired surface pressure (gray-highlighted region). Upon reaching the target pressure, a continuous but slower increase in the barrier position is observed due to the fact that the barriers are still closing, indicating a decrease in the area per molecule. This confirms a rearrangement of the diluent ligand molecules. This behavior is likely associated with the increased molecular freedom and reorientation of the ligand molecules at the liquid–gas interface. Due to the higher surface tension, the molecules tend to adopt a more thermodynamically stable conformation.

**Figure 5:**
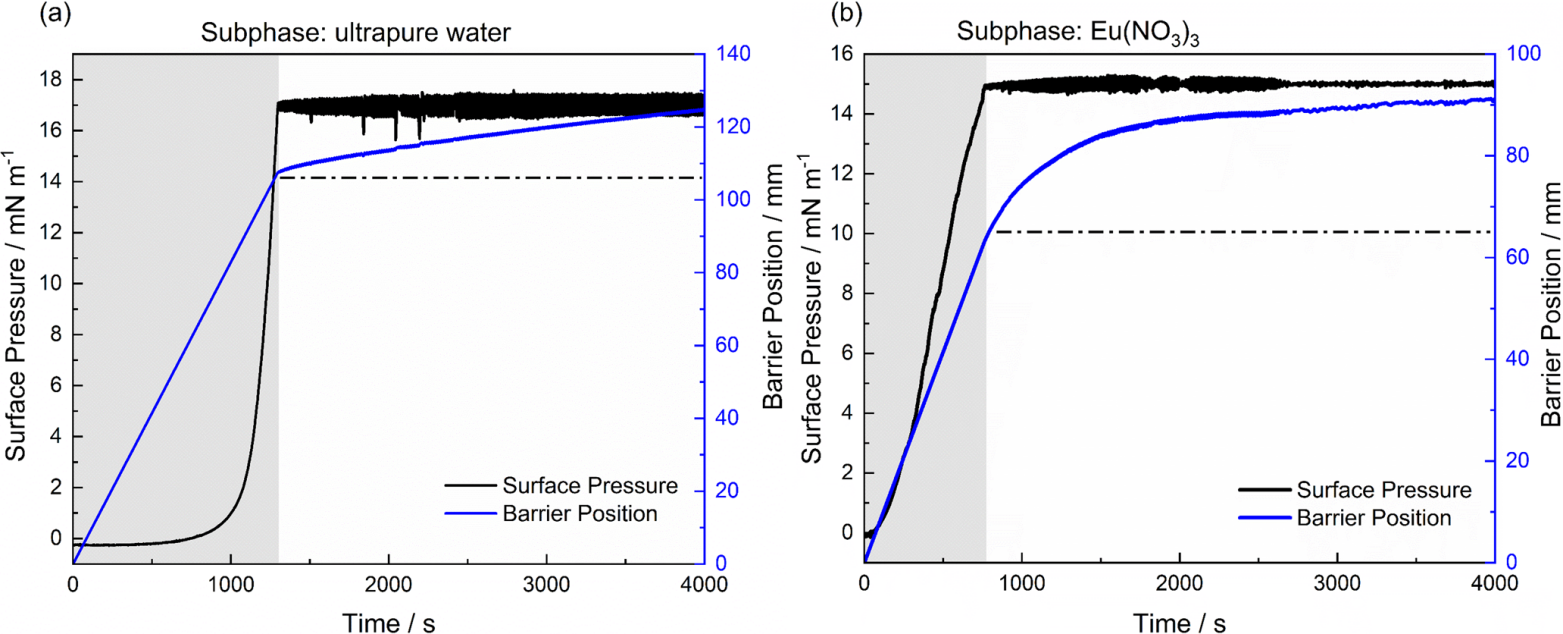
Surface pressure stability tests of dion at the surface of (a) water and (b) in saturated europium nitrate at $2\times 10^{-5}$ mol L^–1^.

On the other hand, interestingly a more pronounced movement of the barriers occurs in the range of ∼800–2000 s in Figure 5b, which may be associated with significant reorganization of the dion ligands as a result of coordination with Eu­(III) ions. After coordination, the film becomes more stable. Up to 2650 s, there is a slight movement, as expected from the isotherm profile, since the G-L phase transition occurs at a higher molecular area, as shown in Figure 4b. This result confirms the effective coordination between the carboxylate groups and subphase ions, suggesting that film compression hinders the possible reorientations of the dion molecules. As favored by the closure of the barriers, the minimum displacement, especially in the range of 2650–4000 s, indicates that the interfacial complex reaches higher stability.

Two cycles of monolayer compression and decompression were carried out at a speed of 10 mm min^–1^ to investigate the effects of the subphase composition on the molecular interactions (Figure 6). The intermolecular interactions in the dion film display more reversibility when spread on an aqueous subphase, since a higher surface pressure is defined at 20 mN m^–1^. In the absence of europium nitrate ions, attractive and repulsive forces are more easily disrupted, contributing to the increased film reversibility (Figure 6a).

**Figure 6:**
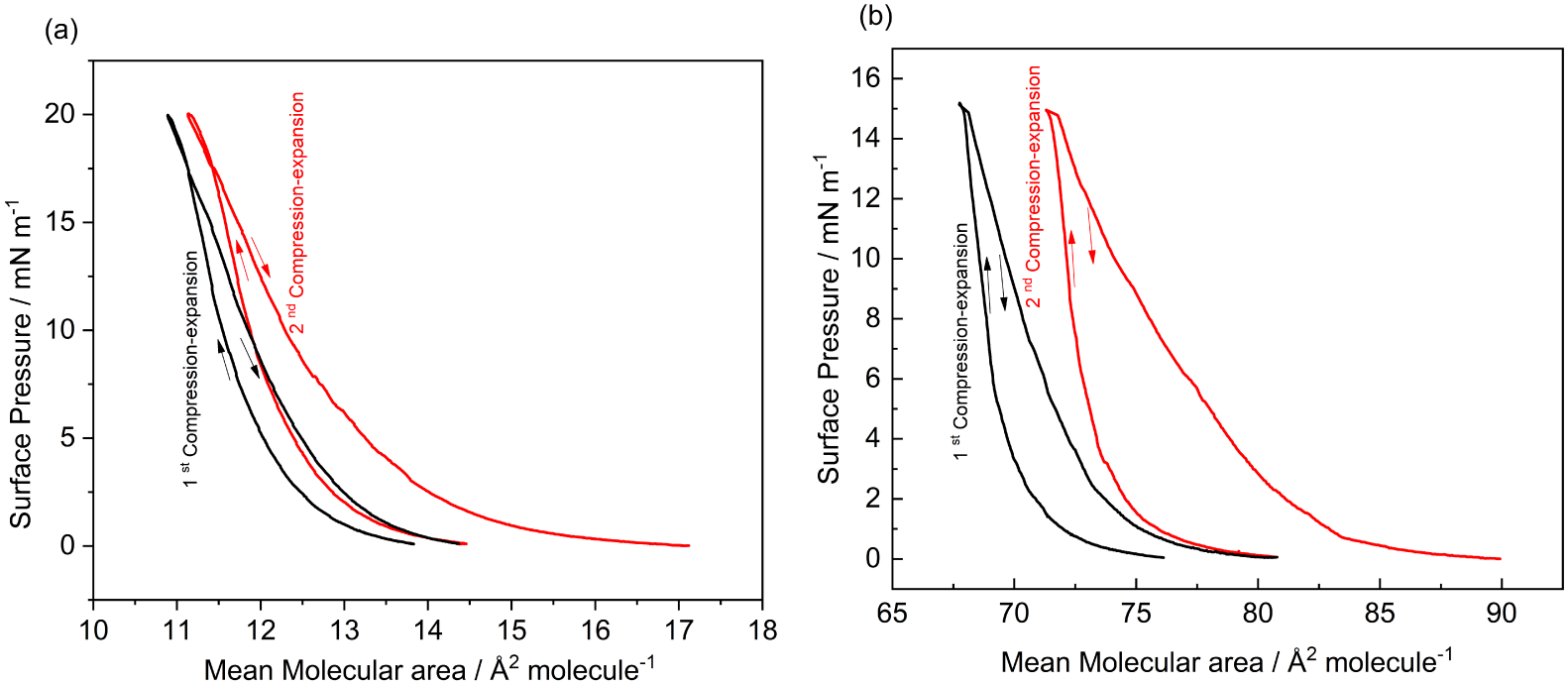
Hysteresis test of the π × *A* isotherms of the dion film on (a) a water subphase and (b) saturated europium nitrate at 2 × 10^–5^ mol L^–1^. Recording of two complete compression and decompression cycles.

The profiles of the hysteresis film at a fixed surface pressure of 15 mN m^–1^ monitored in the subphase saturated with europium nitrate 2 × 10^–5^ mol L^–1^ (Figure 6b) show that the formation of the complex does not lead to the reversibility of the interactions observed for the neat ultrapure water subphase, possibly because the complex remains stable, i.e., the carboxylate and europium ion interaction does not break down easily in the process of opening and closing the barriers. Under these conditions, there is evidence that the organization of the ligand occurs simultaneously with the coordination of its carboxylate groups to the Eu­(NO_3_)_3_·5H_2_O salt present in the subphase at the Liquid–Gas (L–G) interface.

Studies of how molecular organization and inter- and intramolecular interactions influence the spectroscopic properties of interfacially assembled films are crucial for understanding their functional behavior. *In situ* photoluminescence measurements were carried out by monitoring the spectral profile of the luminescent films in which energy transfer from the coordinated ligand to the metallic ion in the subphase allows for elucidation of excitation, energy transfer, and emission mechanisms. Notably, *in situ* luminescence measurements of Langmuir films remain scarcely explored in the literature. Therefore, photoluminescence spectroscopy measurements under ultraviolet excitation were carried out *in situ* in the Langmuir–Blodgett tank (UV-PLS *in situ*) and registered in a real-time during film formation and complexation.

The emission spectra presented in Figure 7 exhibit the characteristic 4f–4f intraconfigurational transitions of the Eu­(III) ion (^5^D_0_ → ^7^F_J_, with *J* = 0–4). The intensity of these transitions, particularly the hypersensitive ^5^D_0_ → ^7^F_2_ band, increases significantly with monolayer compression. This behavior is indicative of the formation of coordination complexes between the Eu­(III) ions and ligands at the interface. As shown in Figure S4, the ^5^D_0_ → ^7^F_2_ emission intensity increases gradually and almost linearly as the barrier position is shifted from 0 to 120 mm (decreasing molecular area), due to the higher concentration of Eu^3+^ ions at the surface as the film is compressed.

**Figure 7:**
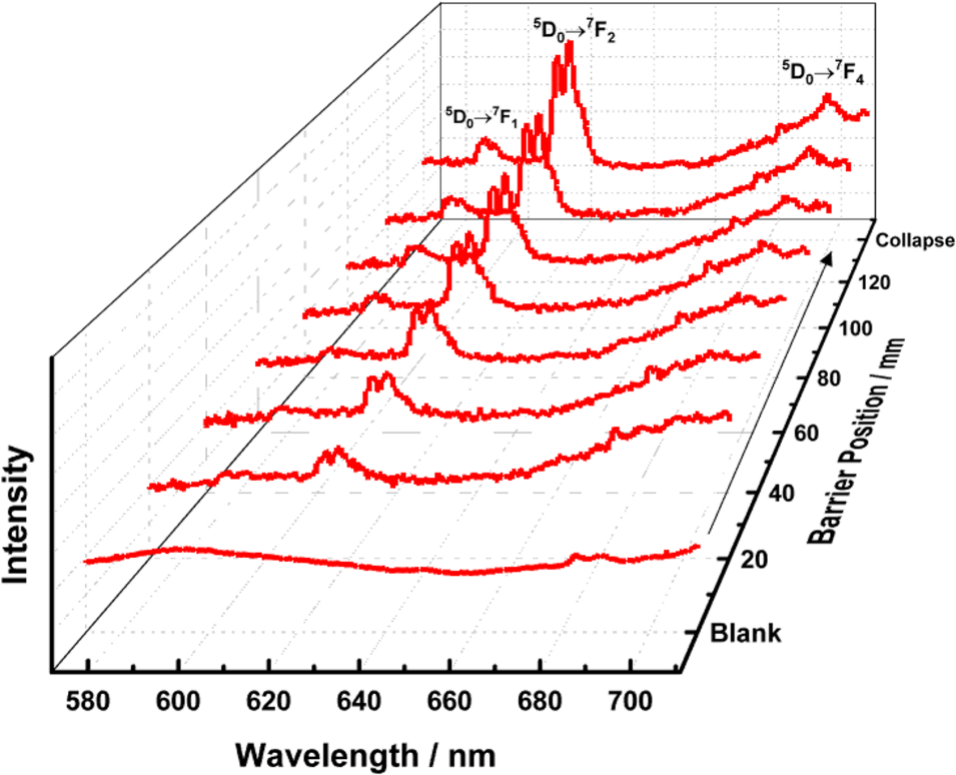
Normalized emission spectra at λ_exc_ = 325 nm recorded *in situ* in the Langmuir–Blodgett cell at 298 K were monitored as a function of the displacement of the movable barriers.

Eu­(III) ions, present in the aqueous subphase, interact with ligands organized in the monolayer, whose packing is modulated by the barrier movement. In the absence of coordination, the interfacial concentration of Eu­(III) would remain constant regardless of the monolayer compression. However, the progressive increase in emission intensity suggests that the formation of luminescent complexes is favored as the ligand density increases. Therefore, monolayer compression enhances ligand proximity, promotes coordination with Eu­(III) ions, and results in a greater accumulation of emissive species at the interface.

Z-Type LbL films were obtained using a descent and an ascent deposition speed of 70 and 10 mm min^–1^, respectively. Under these conditions, successful transfer of the dion Langmuir film spread on a europium nitrate subphase onto a glass substrate was achieved, resulting in a four-monolayer deposition, here denoted as the LB4 film.

Figure 8 displays the luminescence spectra for LB4 films. The region related to excitation transitions between 250 and 500 nm is dominated by intense broad bands, assigned to the ligands.^29,30,34^ The emission spectra monitored at different wavelengths exhibit a similar profile and intensities, dominated by the hypersensitive transition ^5^D_0_ → ^7^F_2_ with a maximum at 622 nm when it is monitored at 323 nm. The lines attributed to 4f–4f intraconfigurational transitions, ^5^D_0_ → ^7^F_J_ with *J* = 1–4, are characteristics of the Eu­(III) ion. The ^5^D_0_ → ^7^F_2_ transition line, when more intense than the one attributed to the ^5^D_0_ → ^7^F_1_ transition, indicates that the local chemical environment around the Eu­(III) ions is of low symmetry. The higher values of ^5^D_0_ → ^7^F_2_ and ^5^D_0_ → ^7^F_1_ intensity ratios suggest a strong mixture between the *f* and *d* Eu­(III) ion orbitals caused by the strong bond between the central ion and ligands.^29,30^

**Figure 8:**
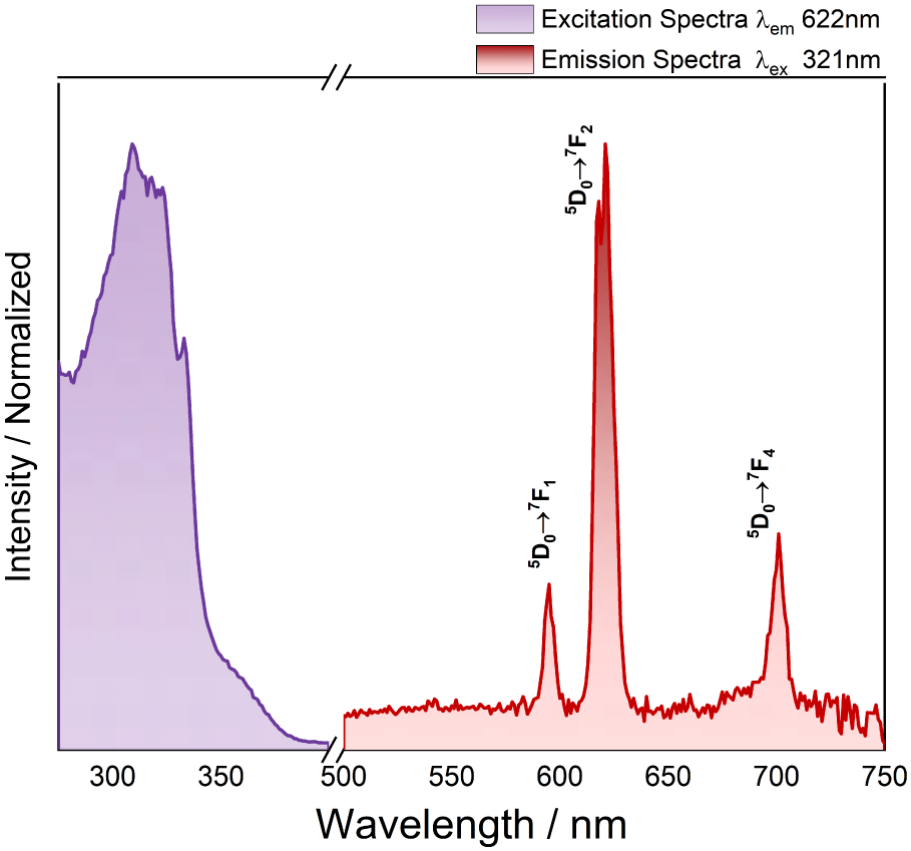
Excitation and emission spectra of the LB4 film at 298 K, monitored at ^5^D_0_ → ^7^F_2_ λ_em_ = 622 nm and λ_exc_ = 321 nm, respectively.

## 4 Conclusions

We synthesized novel coordination complexes to explore the excitation and photoemission mechanisms in bulk materials, Langmuir monolayers, and Langmuir–Blodgett (LB) films. The dion ligand demonstrated strong coordination with Ln­(III) ions, effectively serving as an antenna by transferring energy to the emissive level of the europium ion. The use of a saturated subphase notably enhances interactions between the dion alkyl chains and Eu­(III), promoting the formation of a well-ordered and stabilized monolayer. Such an improved structural arrangement is confirmed here by surface pressure–area isotherms and film stability data. Furthermore, *in situ* photoluminescence data indicate coordination of the ligand to Eu­(III) at the air–water interface, underscoring the efficiency of the self-assembly process. Changes in the ^5^D_0_ → ^7^F_2_ transition suggest variation in the local symmetry around the Eu­(III) ion upon barrier compression. The LB films displayed high homogeneity, evidenced by near-unity transfer ratios, alongside strong luminescence intensity. Altogether, the dion acts both as a surfactant and a photoactive antenna, enabling the fabrication of functional luminescent LB films. These findings emphasize the potential of LB films as versatile platforms for fundamental research and practical applications aimed at improving the performance of optical and electronic devices through molecularly organized systems.

**Highlights**

•The ligand 6-dodecyloxy-2-naphthoic acid (dion) acts as an efficient antenna ligand for obtaining luminescent Eu­(III) complexes.

•*In situ* luminescence studies were conducted during interfacial coordination in a Langmuir trough.

•The ^5^D_0_ → ^7^F_2_emission intensity rises nearly linearly with decreasing molecular area, due to the increased Eu­(III) surface concentration upon film compression.

## Supplementary Material



## Notes

^1^H NMR spectrum; vibration modes (cm^–1^) of dion and the complexes [Eu­(dion)_3_(H_2_O)­(DMSO)] and [Gd­(dion)_3_(H_2_O)­(DMSO)]; phosphorescence spectrum of [Gd­(dion)_3_(H_2_O)­(DMSO)] at 77 K; calculation of the intensity parameters Ω_2_, Ω_4_, and *R*_02_; compressibility modulus and emission spectra profile as a function of hypersensitivity transition and barrier position (PDF)
