# Supplementary material for: In Situ Luminescence of Self-Assembled Eu(III)-Naphthoic Acid Complex in Langmuir and LB Films
Source: Langmuir. 2025 Oct 9;41(41):27968–76. doi: 10.1021/acs.langmuir.5c03763 (PMC12548350; doi:10.1021/acs.langmuir.5c03763)
Supplement: Supplementary file 1 [file la5c03763_si_001.pdf]

In Situ Luminescence of Self-Assembled Eu(III)-Naphthoic Acid Complex in Langmuir and LB films

Sofia Sestito Dias[a], Maria Izabel Xavier Scapolan[a], Wilson Aparecido de Oliveira[a,c], Rhayane Margutti Rocha[b], Higor Henrique de Souza Oliveira[d], Marian Rosaly Davolos[b], Eduard Westphal[a,c], Renata Danielle Adati[a]

[a]Academic Department of Chemistry and Biology, Universidade Tecnológica Federal do Paraná (UTFPR), Curitiba, PR, Brazil

[b]Department of Analytical, Physical-Chemistry and Inorganic Chemistry, São Paulo State University (Unesp), Institute of Chemistry, Araraquara, SP, Brazil.

[c]Department of Chemistry, Universidade Federal de Santa Catarina, Florianópolis, Brazil

[d]IFSP - Instituto Federal de Educação, Ciência e Tecnologia de São Paulo, IFSP - *Campus* Matão, Matão-SP, Brazil.

Supporting Material

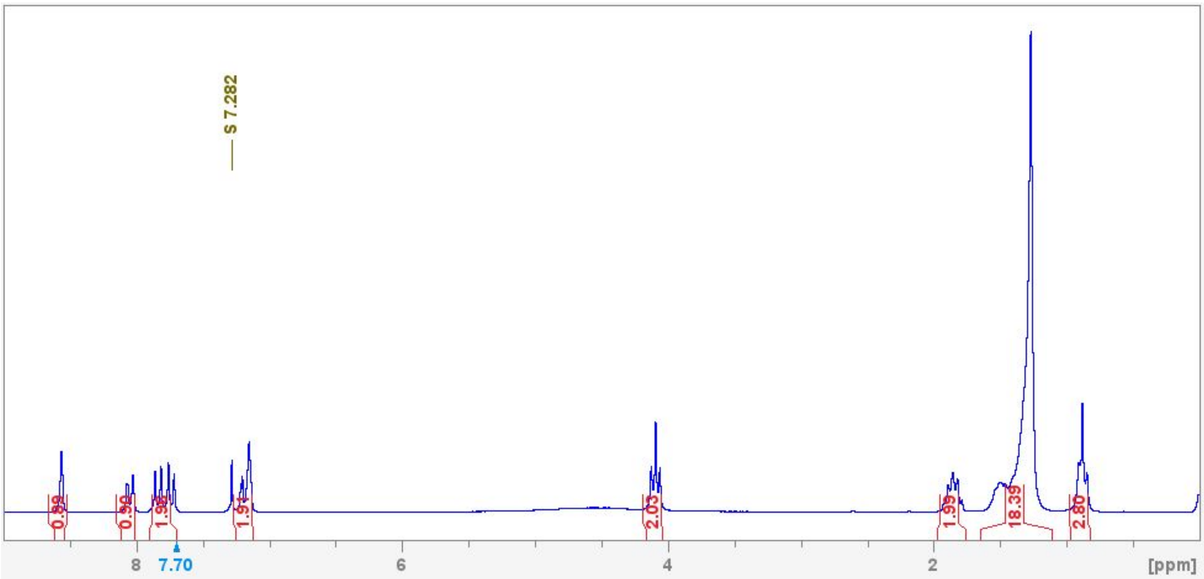

Figure S1. ^{1}H NMR spectrum (400 MHz) of the dion ligand in $CDCl_3$, with tetramethylsilane (TMS) as an internal standard

Table S1. Vibration modes (cm^{-1}) of dion and the complexes $[Eu(dion)_3(H_2O)(DMSO)]$ and $[Gd(dion)_3(H_2O)(DMSO)]$

Attribution attempt	dion	$[Eu(dion)_3(H_2O)(DMSO)]$	$[Gd(dion)_3(H_2O)(DMSO)]$
v_s (O-H)	3448	3448	3440
v_s (C-H)$_{aromático}$	3448	3448	3440
v_s (C=C)$_{aromático}$	1475	1500	1500
v_s (C-O)	1305	1228	1228
v_s (C=O)	1681-1627	1635-1538	1633-1538
v_s (C-H)$_{alifática}$	2931-2856	2931-2856	2921-2854
(Ln-O)	-	450-400	450-400

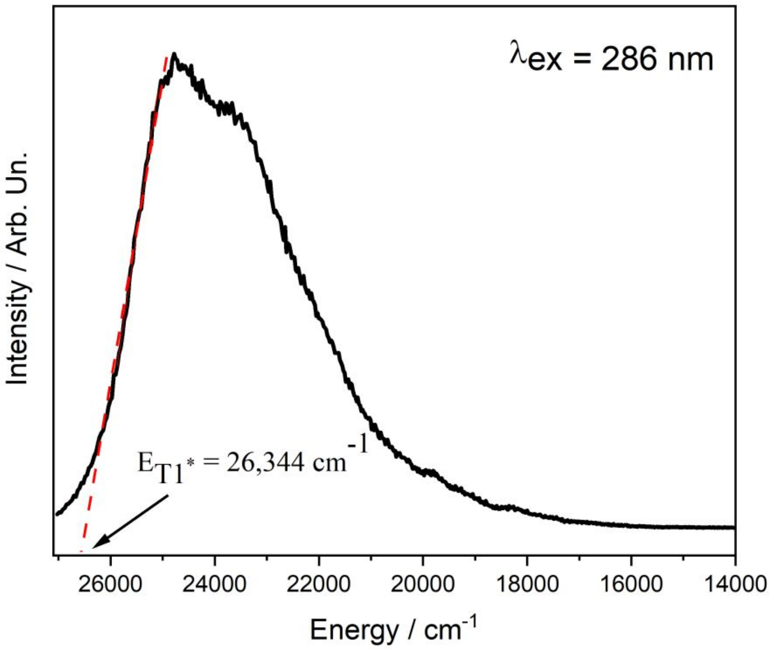

Figure S2. Phosphorescence spectrum of $[Gd(dion)_3(H_2O)(DMSO)]$ complex at 77K.

Calculation of intensity parameters Ω_2, Ω_4 and R_{02}

The Judd-Ofelt intensity parameters, Ω_2 and Ω_4 [35,36] express information about the ligand field and the local symmetry around the Eu(III) ion (Ω_2) and long-range effects and also symmetry (Ω_4) [28, 29]. These parameters can be determined experimentally from the emission intensity from the transitions $^5D_0\rightarrow{}^7F_1$ ($I_{0\rightarrow1}$), $^5D_0\rightarrow{}^7F_2$ ($I_{0\rightarrow2}$), and $^5D_0\rightarrow{}^7F_4$ ($I_{0\rightarrow4}$) calculated by the integrated areas of the lines assigned to the respective transitions.

In the emission spectra of Eu(III) ions, the emission intensity from the transition $^5D_0\rightarrow{}^7F_J$ ($I_{0\rightarrow J}$) is given by Equation (1), where $\sigma_{0\rightarrow J}$ is the energy (cm^{-1}) of the barycenter of the line attributed to the transition $^5D_0\rightarrow{}^7F_J$, $A_{0\rightarrow J}$, is the Einstein spontaneous emission coefficient of the respective transition and N_0 is the population of the $^5D_0$ emission level [28, 29]. From the emission spectra of the complexes containing the Eu(III) ion obtained at room temperature, it was possible to determine the parameters of experimental intensities Ω_λ (λ = 2 and 4) of the transitions $^5D_0\rightarrow{}^7F_{2,4}$ and the intensity parameter R_{02}, which provides information on the *J*

mixing effect associated with the $^5D_0 \rightarrow {}^7F_0$ transition. The areas under the curves as well as the energies of the transitions were calculated using the LUMPAC software.

$$I_{0\rightarrow J} = \sigma_{0\rightarrow J} A_{0\rightarrow J} N_0 \qquad \textbf{(1)}$$

In the case of the reference transition $^5D_0 \rightarrow {}^7F_1$, the values of $I_{0\rightarrow 1}$ and $\sigma_{0\rightarrow 1}$ can be obtained from the emission spectrum. The value of $A_{0\rightarrow 1}$ is expressed by Equation 2, where *n* represents the refractive index of the medium.

$$A_{0\rightarrow 1} = 0{,}31.10^{-11}\, n^3 (\sigma_{0\rightarrow 1})^3 \qquad \textbf{(2)}$$

Population N_0 is the same for all $^5D_0 \rightarrow {}^7F_J$ transitions, as these transitions occur from the same $^5D_0$ emitter level. Therefore, it is possible to establish a relationship between the intensities and energy of the $^5D_0 \rightarrow {}^7F_J$ transitions (J = 2 or 4) with the intensity and energy of the reference transition $^5D_0 \rightarrow {}^7F_1$, as shown in Equation 3 [28]. The values of $I_{0\rightarrow J}$ and $\sigma_{0\rightarrow J}$ for the $^5D_0 \rightarrow {}^7F_J$ transitions (J = 2 or 4) can be obtained from the emission spectrum, being then possible to determine the values of $A_{0\rightarrow 2}$ and $A_{0\rightarrow 4}$ by Equation 3.

$$A_{0\rightarrow J} = \frac{I_{0\rightarrow J}}{I_{0\rightarrow 1}} \frac{\sigma_{0\rightarrow 1}}{\sigma_{0\rightarrow J}} \cdot A_{0\rightarrow 1} \qquad \textbf{(3)}$$

With the values of the spontaneous emission coefficient $A_{0\rightarrow 2}$ and $A_{0\rightarrow 4}$, it is possible to calculate the parameters of Judd-Ofelt intensity, Ω_2 and Ω_4 from Equations 4 and 5, respectively [38,39].

$$\Omega_2 = \frac{3\hbar c^3 A_{0\rightarrow 2}}{4e^2 (\sigma_{0\rightarrow 2})^3 [n\,(n^2+2)^2/9]\,\langle {}^7F_2 \| U^{(4)} \| {}^5D_0 \rangle^2} \qquad \textbf{(4)}$$

$$\Omega_4 = \frac{3\hbar c^3 A_{0\rightarrow 4}}{4e^2 (\sigma_{0\rightarrow 4})^3 [n\,(n^2+2)^2/9]\,\langle {}^7F_4 \| U^{(4)} \| {}^5D_0 \rangle^2} \qquad \textbf{(5)}$$

In equation 6, $\hbar = h/2_{\pi}$, where *h* is Planck's constant, and the elementary charge, *c* is the speed of light in vacuum, and the term $\langle {}^7F_J \| U^{(J)} \| {}^5D_0 \rangle^2$ corresponds to the diagonalized elements of the squared matrix, with values of 0.0032 and 0.0023 for Ω_2 and Ω_4, respectively. Analyzing Equation 4, it is observed that the Ω_2 parameter is proportional to the Einstein spontaneous emission coefficient $A_{0\rightarrow 2}$, which depends on the intensity ratio 1 $I_{0\rightarrow 2}/I_{0\rightarrow 1}$. Therefore, Ω_2 is proportional to the parameter R_{21}, both related to the local symmetry around the Eu(III) ion].

Other parameters calculated from the emission spectrum and lifetime value were the radioactive (A_{rad}) and non-radiative (A_{nrad}) decay rates, the quantum efficiency (η) and the intensity parameter R_{02}, which provides information about the *J mixing effect* associated with the intensity of the $^5D_0 \rightarrow {}^7F_0$ transition relative to the $^5D_0 \rightarrow {}^7F_2$ transition.

Once the emitter level is filled, the radiative (A_{rad}) and non-radiative (A_{nrad}) decay rates define the quantum efficiency η for the $^5D_0$ emitter level, as shown in equation 6.

The radiative decay rate (A_{rad}) is obtained by summing the $A_{0\rightarrow J}$ Einstein spontaneous emission coefficients of all $^5D_0 \rightarrow {}^7F_J$ transitions, which can be calculated by Equations 2 and 3. Through the A_{rad} value, the rate of radiative decay non-radiative decay (A_{nrad}) can be calculated using Equation 6, which relates the experimentally measured lifetime to the A_{rad} and A_{nrad} decay rates[39].

$$\frac{1}{\tau} = A_{rad} + A_{nrad} \qquad \textbf{(6)}$$

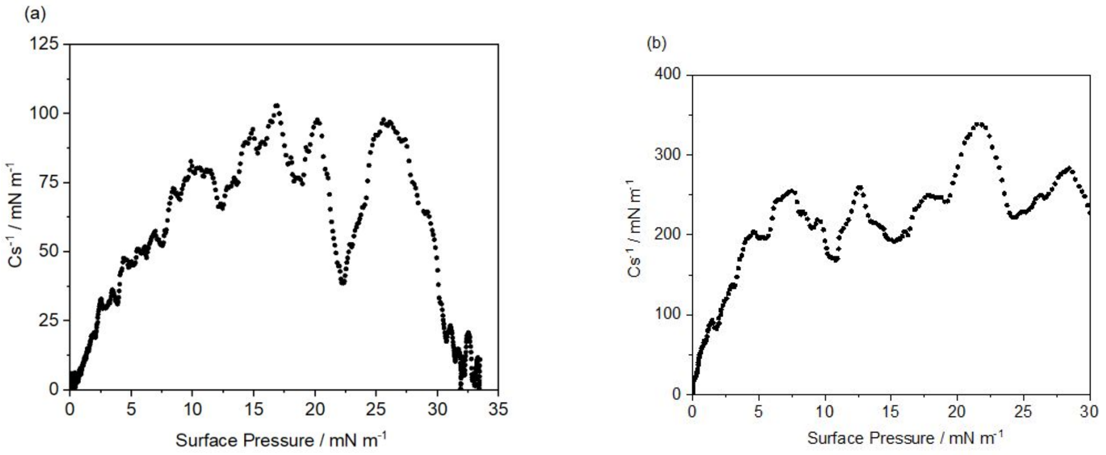

Figure S3. Compressibility modulus (Cs^{-1}) as a function of surface pressure for dion monolayer on (a) water subphase and (b) saturated europium nitrate at 2×10^{-5} mol L^{-1}.

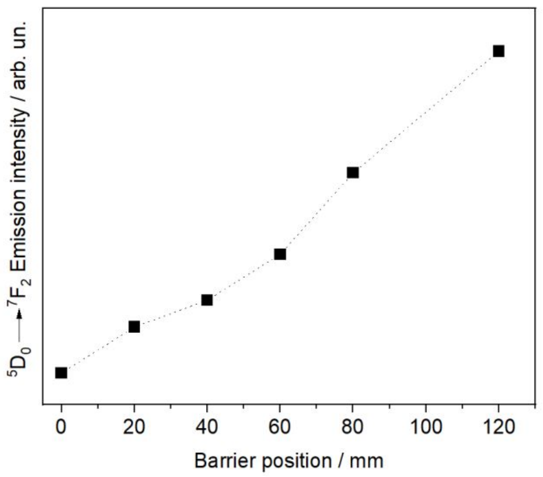

Figure S4. $^5D_0 \rightarrow {}^7F_2$ normalized emission spectra obtained as a barrier position recorded *in situ* in the Langmuir-Blodgett.
